# Postoperative Management After Risk-Reducing Salpingo-Oophorectomy for Hereditary Breast and Ovarian Cancer Syndrome: A Narrative Review on Balancing Oncologic Benefit and Quality of Life

**DOI:** 10.7759/cureus.101823

**Published:** 2026-01-19

**Authors:** Hiroaki Ishida, Akiko Takashima

**Affiliations:** 1 Department of Obstetrics and Gynecology, Toho University Sakura Medical Center, Sakura, JPN

**Keywords:** hboc, hrt, ovarian cancer, rrso, stic

## Abstract

Risk-reducing salpingo-oophorectomy (RRSO) is the most effective intervention for reducing ovarian cancer risk in women with hereditary breast and ovarian cancer syndrome (HBOC) associated with pathogenic *BRCA1* and *BRCA2* variants. RRSO has been shown to markedly reduce the risk of ovarian cancer and to confer a protective effect against breast cancer, thereby contributing to improved overall survival. However, when performed before natural menopause, RRSO induces abrupt menopause, which is associated with vasomotor symptoms, sexual dysfunction, accelerated bone loss, adverse cardiovascular changes, mood disturbances, and reduced quality of life. Recent meta-analyses have also demonstrated an increased risk of all-cause mortality among women undergoing oophorectomy before the age of 45 years without hormone replacement therapy.

Serous tubal intraepithelial carcinoma (STIC), a recognized precursor of high-grade serous carcinoma, is occasionally identified in specimens obtained during RRSO and is associated with an increased risk of subsequent primary peritoneal cancer, warranting tailored postoperative surveillance. International guidelines emphasize comprehensive management following RRSO, including symptom-directed therapies, hormone replacement therapy for eligible patients, cardiovascular and bone health monitoring, and routine breast cancer surveillance with contrast-enhanced magnetic resonance imaging. While no additional gynecologic screening is recommended for patients without STIC, those with STIC require follow-up protocols similar to ovarian cancer surveillance. Emerging evidence also supports prophylactic salpingectomy with delayed oophorectomy as a staged risk-reduction strategy that preserves ovarian function, mitigates menopause-related morbidity, and maintains preventive benefits.

This narrative review summarizes current evidence regarding the oncologic benefits and adverse health consequences of RRSO, the clinical significance of STIC, international postoperative care recommendations, and evolving preventive strategies. Individualized, multidisciplinary postoperative management is essential to optimize long-term health outcomes and quality of life in women undergoing RRSO.

## Introduction and background

Hereditary breast and ovarian cancer syndrome (HBOC) is a genetic cancer predisposition characterized by pathogenic variants in the breast cancer susceptibility genes *BRCA1* or *BRCA2*. Individuals carrying these variants have an increased risk of developing breast cancer, ovarian cancer, and other malignancies [1]. The *BRCA* genes play a critical role in DNA repair, and mutations in* BRCA1* or *BRCA2* impair their tumor-suppressive function, thereby increasing cancer susceptibility. These mutations are frequently associated with a family history of cancer and serve as important indicators in hereditary cancer risk assessment. Approximately 13-18% of ovarian cancer cases are attributable to pathogenic *BRCA* variants [2,3].

Risk-reducing salpingo-oophorectomy (RRSO) is currently the most effective preventive strategy for ovarian cancer in patients with HBOC. RRSO has been shown to reduce the risk of ovarian cancer by approximately 80% and breast cancer by 50%, while also improving overall survival [4-6]. Because no effective screening modality for the early detection of ovarian cancer has been established, RRSO remains the cornerstone of ovarian cancer prevention. In Japan, RRSO became covered by the national insurance system in April 2020 for breast cancer patients with HBOC, leading to an increase in the number of procedures performed nationwide [7].

Current clinical guidelines recommend performing RRSO between 35 and 40 years of age for *BRCA1 *mutation carriers and between 40 and 45 years of age for* BRCA2* mutation carriers [8]. However, bilateral oophorectomy in premenopausal women induces abrupt surgical menopause and is associated with decreased bone mineral density. Additionally, the incidental detection of serous tubal intraepithelial carcinoma (STIC), a precursor lesion of high-grade serous ovarian carcinoma, in RRSO specimens is associated with an increased risk of subsequent primary peritoneal carcinoma [9]. Therefore, based on gynecologic oncology principles, the management of premature menopause, and established HBOC guidelines, the development of a comprehensive postoperative care strategy following RRSO is essential.

The primary objective of this narrative review is to synthesize current evidence regarding postoperative management after RRSO in women with hereditary breast and ovarian cancer syndrome, with particular emphasis on balancing oncologic risk reduction and long-term quality of life. This review focuses on key postoperative challenges, including surgically induced menopause, hormone replacement therapy considerations, the clinical significance of serous tubal intraepithelial carcinoma, and stratified postoperative surveillance strategies. The intended audience includes gynecologic oncologists, general gynecologists, genetic counselors, and other clinicians involved in the multidisciplinary care of patients with hereditary cancer syndromes.

## Review

Methodology

This article was designed as a narrative review rather than a formal systematic review. A targeted literature search was conducted using PubMed and major guideline repositories to identify relevant meta-analyses, systematic reviews, large observational cohort studies, and international clinical practice guidelines published primarily within the past 15 years. Studies were prioritized based on clinical relevance, methodological rigor, and impact on postoperative management after RRSO. Given the narrative nature of this review, formal inclusion and exclusion criteria, quantitative data pooling, and structured risk-of-bias assessments were not performed. When available, evidence certainty was described based on the original authors’ assessments and guideline consensus statements, and limitations related to evidence quality were explicitly acknowledged.

Evidence of Cancer Risk Reduction and Postoperative Quality of Life After RRSO

Clinical trials and observational studies demonstrating the cancer risk-reducing effects of RRSO: For carriers of pathogenic *BRCA1* and* BRCA2 *variants, RRSO is the only intervention with proven efficacy in substantially reducing the risk of ovarian cancer. Recent meta-analyses have demonstrated that RRSO significantly decreases ovarian cancer incidence and improves overall survival (Table 1) (Certainty of evidence: High) [10]. As noted above, no effective surveillance strategy for the early detection of ovarian cancer has been established, and RRSO remains the sole preventive measure with confirmed oncologic benefit in this population. Furthermore, a 2019 meta-analysis reported a significant reduction in newly diagnosed breast cancer among both unaffected *BRCA* variant carriers and those with a prior history of breast cancer following RRSO [11]. Although ovarian cancer risk reduction remains the primary indication for the procedure, the associated decrease in breast cancer risk represents valuable information for patients considering RRSO.

**Table 1:** Effect of RRSO on overall survival and cancer-specific mortality HR: Hazard Ratio, RRSO: Risk-Reducing Salpingo-Oophorectomy, BRCA: Breast Cancer Susceptibility Gene (*BRCA1* / *BRCA2*), HGSC: High-Grade Serous Carcinoma The table was created based on Reference [10].

Outcome	HR (95% CI)	P-value	Studies (n)	Interpretation
Overall survival (all)	0.32 (0.19–0.54)	<0.001	3 studies (2548)	Improved survival with RRSO
Overall survival *(BRCA1*)	0.30 (0.17–0.52)	<0.001	3 studies	Improved survival
Overall survival (*BRCA2*)	0.44 (0.23–0.85)	0.01	2 studies	Improved survival
HGSC mortality (all)	0.06 (0.02–0.17)	<0.0001	3 studies (2534)	Reduced HGSC mortality
HGSC mortality (*BRCA1*)	0.10 (0.02–0.41)	0.001	2 studies	Reduced mortality
HGSC mortality (*BRCA2*)	Not estimable	–	–	Insufficient data
Breast cancer mortality (all)	0.58 (0.39–0.88)	0.009	7 studies (7198)	Reduced mortality
Breast cancer mortality (*BRCA1*)	0.45 (0.30–0.67)	<0.0001	4 studies	Reduced mortality
Breast cancer mortality (*BRCA2*)	0.88 (0.42–1.87)	0.75	3 studies	No significant difference

Reports on overall survival, quality of life, and menopausal symptoms after RRSO: While RRSO is highly effective in reducing ovarian cancer risk in women with pathogenic *BRCA1 *and *BRCA2* variants, the procedure induces abrupt surgical menopause when performed in premenopausal women. Surgical menopause has been associated with shorter survival compared with natural menopause, as well as substantial short- and long-term health consequences. Women who undergo bilateral oophorectomy before the age of 45 years have a significantly increased risk of all-cause mortality, with a reported 1.67-fold increase (95% confidence interval (CI): 1.16-2.40, p = 0.006) (Table 2). Elevated mortality rate has also been reported among women undergoing salpingo-oophorectomy before age 45 in the absence of subsequent hormone replacement therapy (HRT) (Table 3) [12].

**Table 2:** Overall and stratified survival after bilateral oophorectomy (compared with referent women); age at prophylactic oophorectomy The table was created based on Reference [12].

Age at surgery	Risk of all-cause mortality hazard ratio (95% CI)	p-value
<45 years	1.67 (1.16–2.40)	0.006
45–50 years	1.02 (0.78–1.32)	0.90
>50 years	0.90 (0.68–1.19)	0.46

**Table 3:** Effect of estrogen therapy on mortality after bilateral oophorectomy before age 45 The table was created based on Reference [12].

Group	Mortality rate hazard ratio (95% CI)	Interpretation
Oophorectomy <45 years + No HRT until 45	1.93 (1.25–2.96)	Increased mortality
Oophorectomy <45 years + HRT until 45	1.27 (0.67–2.39)	No significant difference

From a women’s health perspective, several important considerations arise following RRSO. Estrogen deficiency resulting from surgical menopause contributes to vasomotor symptoms, cardiovascular disease, dyslipidemia, cognitive decline, osteoporosis, and sexual dysfunction, all of which negatively affect quality of life (QOL). Although fracture risk does not appear to increase within a median follow-up of 6.9 years among *BRCA1 *and *BRCA2* carriers who undergo RRSO, the prevalence of osteoporosis diagnoses is higher in this population (Table 4) [13].

**Table 4:** Risk of fracture and osteoporosis after prophylactic bilateral salpingo-oophorectomy (RRSO) *BRCA: *Breast Cancer Susceptibility Gene, RRSO: Risk-Reducing Salpingo-Oophorectomy The table was created based on Reference [13].

Outcome	Comparison Group (n)	Adjusted Hazard Ratio	95% CI	Interpretation
Fracture Risk	*BRCA* mutation RRSO (329) vs BO group (3290)	0.80	0.56 – 1.14	Not significant
Fracture Risk	*BRCA* mutation RRSO (329) vs Intact ovaries (3290)	1.02	0.65 – 1.61	Not significant
Osteoporosis Risk (DEXA subset)	*BRCA* mutation RRSO (152) vs BO group (761)	1.60	1.00 – 2.54	Significantly higher
Osteoporosis Risk (DEXA subset)	*BRCA* mutation RRSO (152) vs Intact ovaries (335)	2.49	1.44 – 4.28	Significantly higher

Menopausal symptoms have been reported in the early postoperative period. Within three months following RRSO, the prevalence of hot flashes increases from 5.3% to 56.2%, while the prevalence of night sweats rises from 20.2% to 47.2% (Table 5) [14].

**Table 5:** Three-month post-RRSO prevalence of hot flashes and night sweats RRSO: Risk-Reducing Salpingo-Oophorectomy The table was created based on Reference [14].

Outcome	Pre-RRSO (n=95)	3 Months Post-RRSO (n-95)	Notes
Hot flushes	5.3%	56.2%	Marked increase after RRSO
Night sweats	20.2%	47.2%	Marked increase after RRSO

Management of QOL and Menopausal Symptoms After RRSO

International evidence-based guidelines have systematically summarized recommendations for the management of menopausal symptoms, sleep disturbances, mood disorders, sexual dysfunction, urogenital symptoms, cardiovascular health, and bone health in women undergoing RRSO [15]. These guidelines highlight the importance of comprehensive care both before and after RRSO. Recommended management strategies across these domains are presented in Table 6 [15-17].

**Table 6:** Management of symptoms after premenopausal risk-reducing salpingo-oophorectomy (RRSO) SSRIs: Selective Serotonin Reuptake Inhibitors, HRT: Hormone Replacement Therapy A table was created based on References [15-17].

Symptoms After Premenopausal Risk-Reducing Salpingo-Oophorectomy (RRSO)	Management / Other Notes
Vasomotor symptoms (hot flashes, flushing, sweating)	Kampo medicine, soy isoflavones, SSRIs, HRT (generally not used in breast cancer patients)
Sleep disturbance	SSRIs, hypnotics / sleep-inducing agents
Mood disorders	Antidepressants
Sexual dysfunction	Lubricating jelly; vaginal estrogen therapy may also be considered
Cardiovascular disorders	HRT (generally not used in breast cancer patients), lifestyle counseling
Decreased bone density	Bone density measurement once per year
Cognitive decline	Cognitive decline may increase after surgical oophorectomy performed before age 45

HRT has been shown to improve QOL following RRSO compared with nonuse [18]. For *BRCA *variant carriers without a history of breast cancer, HRT is strongly recommended until approximately 45 years of age to prevent the risks of osteoporosis and cardiovascular disease. In contrast, for patients with a history of breast cancer, HRT is generally contraindicated or should be administered with extreme caution due to concerns regarding cancer recurrence (Certainty of evidence: Low) [15]. In breast cancer survivors, the use of hormone replacement therapy after RRSO remains particularly complex. Limited observational data suggest that short-term HRT may be considered in carefully selected patients, such as those with estrogen receptor-negative disease, severe menopausal symptoms, or substantial risk of osteoporosis or cardiovascular disease. The duration of therapy should generally be minimized, and systemic estrogen use should be approached with caution. Local vaginal estrogen therapy may be considered for urogenital symptoms when non-hormonal options are ineffective. Given the low certainty of available evidence, decisions regarding HRT in this population should be individualized and made through shared decision-making involving oncology specialists.

Pathological Studies on STIC and the Mechanisms of Peritoneal Carcinogenesis

High-grade serous carcinoma (HGSC) of the ovary is now widely believed to originate primarily from STIC arising in the fimbrial end of the fallopian tube [18]. At present, no preoperative screening modality is available for the detection of STIC, and the diagnosis can only be established following surgical removal of the adnexa. A meta-analysis has reported that STIC is defined in approximately 2.8% of specimens obtained during RRSO [19].

A subsequent meta-analysis published in 2022 reported that patients with STIC identified after RRSO have a substantially increased risk of developing primary peritoneal carcinoma, with a cumulative incidence of 10.5% at 5 years and 27.5% at 10 years. In contrast, patients without STIC had markedly lower incidences of peritoneal carcinoma, estimated at 0.3% at 5 years and 0.9% at 10 years (Table 7) [9].

**Table 7:** RRSO: incidence of postoperative peritoneal cancer according to the presence or absence of STIC RRSO: Risk-Reducing Salpingo-Oophorectomy, STIC: Serous Tubal Intraepithelial Carcinoma The table was created based on Reference [9].

Incidence of primary peritoneal cancer after RRSO	5 years after	10 years after
STIC Group	10.5%	27.5%
No-STIC Group	0.3%	0.9%

In Japan, the reported 5-year overall survival rates for stage IA-IC3 epithelial ovarian cancer range from 94.9-84.9% [20], which are comparable to the proportion of patients without peritoneal cancer development among those with STIC detected after RRSO. Additionally, the lifetime risk of ovarian cancer in the general population is approximately 1.1-1.3% [21], a rate similar to the incidence of peritoneal cancer observed in patients without STIC. Collectively, these findings support the need to consider postoperative surveillance strategies based on the presence or absence of STIC.

Although the ESGO-ESMO-ESP consensus conference has published recommendations regarding staging surgery for patients with STIC detected after RRSO [22], the available evidence remains limited, and no standardized postoperative management strategy has been definitively established.

Postoperative Management After RRSO

ESMO international guidelines: The 2023 European Society for Medical Oncology (ESMO) Clinical Practice Guidelines recommend annual contrast-enhanced breast magnetic resonance imaging (MRI) for patients with HBOC [23]. Accordingly, patients who have undergone surgical treatment for breast cancer but have not received contralateral prophylactic mastectomy should continue routine surveillance with contrast-enhanced breast MRI to facilitate early detection.

Patients undergoing RRSO should also receive thorough counseling regarding the short- and long-term health consequences of premature menopause. Although some evidence suggests that HRT may be safe, recent studies indicate that its use may be appropriate primarily for women up to approximately 45 years of age without increasing breast cancer incidence. Beyond this age, HRT may be associated with an increased breast cancer risk; therefore, if considered after RRSO, short-term HRT may be considered only after comprehensive counseling regarding potential risks and benefits. Patients should be informed of the current limitations of evidence supporting HRT use. While HRT is effective in alleviating menopausal symptoms and reducing osteoporosis risk, its effects on cardiovascular and cognitive outcomes remain inconclusive. The ESMO guidelines also state that routine gynecologic surveillance after RRSO is not recommended [23].

Proposed Postoperative Management Strategy

**Figure 1:**
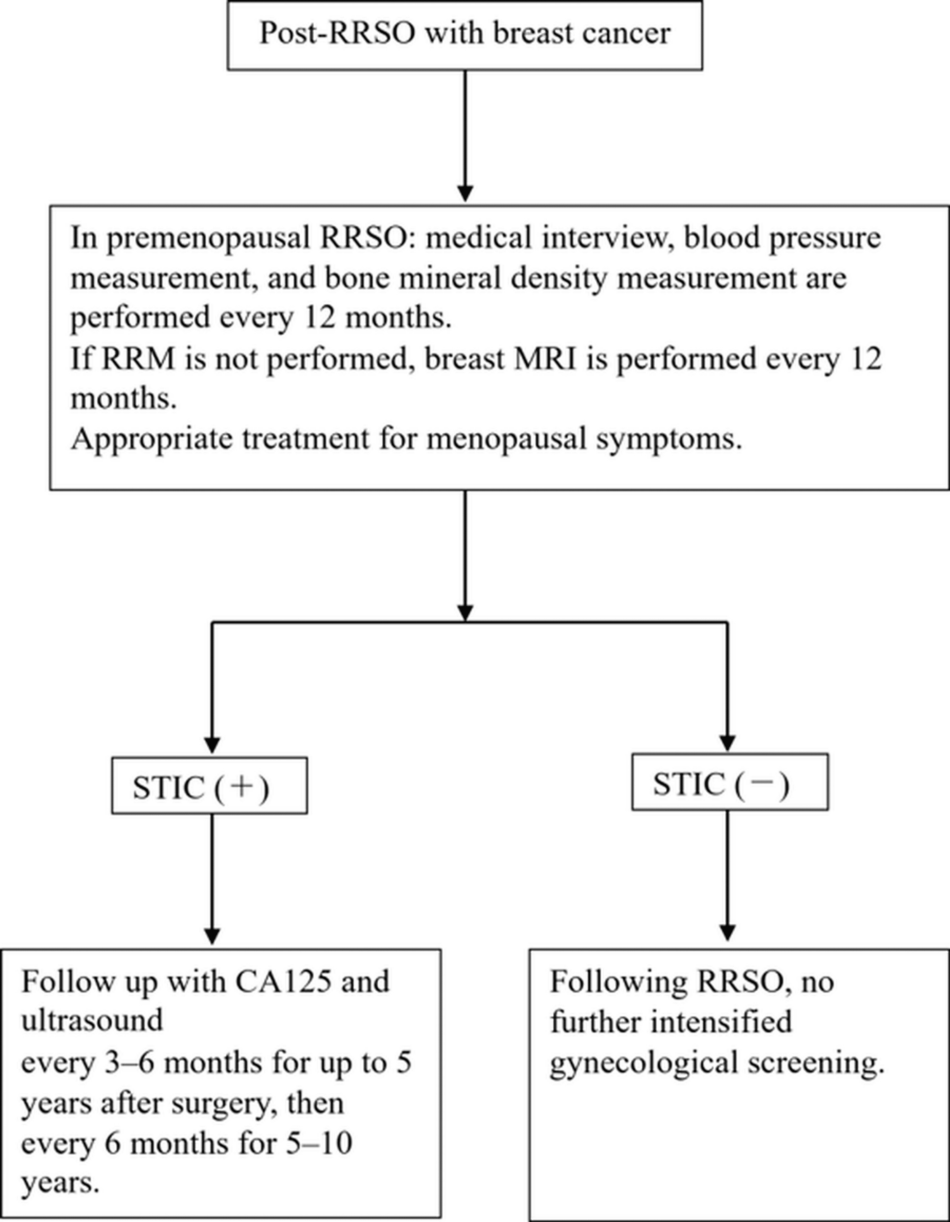
Suggestions for postoperative management of RRSO RRSO: Risk-Reducing Salpingo-Oophorectomy, RRM: Risk-Reducing Mastectomy, MRI: Magnetic Resonance Imaging, STIC: Serous Tubal Intraepithelial Carcinoma Source: Reference [24]

Based on a review of the available literature, we developed a postoperative follow-up flowchart (Figure 1) to guide clinical management after RRSO [24]. In summary, premenopausal women undergoing RRSO should receive annual medical evaluations, including a structured clinical assessment, blood pressure measurement, and bone mineral density testing. For patients who have not undergone a risk-reducing mastectomy, annual contrast-enhanced breast MRI is recommended. Menopausal symptoms should be managed using pharmacologic therapies as clinically indicated [24].

Postoperative surveillance for primary peritoneal carcinoma should be stratified according to the presence or absence of STIC. Although patients with STIC detected after RRSO have a substantially increased risk of developing primary peritoneal carcinoma, there is currently no international consensus guideline defining optimal postoperative surveillance strategies in this population. Recommendations for periodic transvaginal ultrasonography and serum CA-125 measurement in STIC-positive patients are based on extrapolation from ovarian cancer follow-up practices and available observational data rather than high-level evidence. The potential psychological burden and risk of over-surveillance should be carefully considered, and follow-up strategies should be individualized through shared decision-making until prospective data become available. Conversely, for patients without STIC, the risk of developing primary peritoneal cancer is minimal, and no additional gynecologic screening beyond standard postoperative care is recommended after RRSO [24].

Future directions: salpingectomy with delayed oophorectomy as a staged risk-reduction strategy

Salpingectomy has been proposed as a potentially effective strategy for reducing the risk of HGSC. Prophylactic salpingectomy with delayed oophorectomy (PSDO) has emerged as an alternative risk-reduction approach for premenopausal *BRCA1* and *BRCA2* mutation carriers who wish to avoid immediate oophorectomy [25]. PSDO aims to balance oncologic risk reduction through salpingectomy with preservation of ovarian function, thereby maintaining QOL. PSDO consists of a two-stage procedure, with salpingectomy performed first, followed by delayed oophorectomy several years later.

In a clinical study by Steenbeek et al. involving 577 participants, menopause-related QOL was significantly better among women who underwent PSDO compared with those who underwent immediate RRSO. Although HRT improved menopausal symptoms in the immediate RRSO group, outcomes did not reach the levels observed in the PSDO group [25]. The TUBA-WISP II study, currently the largest ongoing trial evaluating this approach, includes women aged 25-40 years who undergo salpingectomy followed by delayed oophorectomy up to five years beyond the recommended age for standard RRSO. The primary endpoint of the trial is the incidence of HGSC, and the trial aims to determine whether PSDO is non-inferior to standard RRSO.

Future research priorities in the postoperative management of patients undergoing RRSO include: prospective and randomized studies evaluating optimal postoperative surveillance strategies, particularly for patients with STIC; long-term studies assessing cardiovascular, cognitive, and metabolic outcomes after premenopausal RRSO; controlled trials examining the safety and efficacy of hormone replacement therapy in specific subgroups, including breast cancer survivors; the development of biomarkers or risk stratification tools to predict primary peritoneal cancer after RRSO.

## Conclusions

RRSO remains the most effective strategy for preventing ovarian cancer in patients with HBOC. However, the substantial physical, psychological, and social consequences associated with surgically induced menopause underscore the need for comprehensive and individualized postoperative management. Multidisciplinary follow-up care is essential for optimizing patient QOL.
